# Fatostatin induces pro- and anti-apoptotic lipid accumulation in breast cancer

**DOI:** 10.1038/s41389-018-0076-0

**Published:** 2018-08-24

**Authors:** Viktor Brovkovych, Yasir Izhar, Jeanne M. Danes, Oleskii Dubrovskyi, Isin T. Sakallioglu, Lauren M. Morrow, G. Ekin Atilla-Gokcumen, Jonna Frasor

**Affiliations:** 1 Department of Physiology and Biophysics, University of Illinois at Chicago, Chicago, IL 60612 USA; 2 Department of Chemistry, University of Buffalo, Buffalo, NY 14260 USA

## Abstract

Given the dependence of cancers on de novo lipogenesis, we tested the effect of fatostatin, a small molecule thought to target this pathway by blocking activation of SREBP transcription factors, in breast cancer cell lines and xenograft tumors. We found that estrogen receptor (ER) positive cells were more sensitive to fatostatin than ER negative cells and responded with cell cycle arrest and apoptosis. Surprisingly, we found that rather than inhibiting lipogenesis, fatostatin caused an accumulation of lipids as a response to endoplasmic reticulum stress rather than inhibition of SREBP activity. In particular, ceramide and dihydroceramide levels increased and contributed to the apoptotic effects of fatostatin. In addition, an accumulation of triacylglycerides (TAGs), particularly those containing polyunsaturated fatty acids (PUFAs), was also observed as a result of elevated diacylglycerol transferase activity. Blocking PUFA-TAG production enhanced the apoptotic effect of fatostatin, suggesting that these lipids play a protective role and limit fatostatin response. Together, these findings indicate that the ability of breast cancer cells to respond to fatostatin depends on induction of endoplasmic reticulum stress and subsequent ceramide accumulation, and that limiting production of PUFA-TAGs may be therapeutically beneficial in specific tumor subtypes.

## Introduction

Increased uptake and anaerobic metabolism of glucose, even in the presence of oxygen (i.e., the Warburg effect), is a well-accepted hallmark of cancer^1^. This is considered an important feature as it provides both energy for cell growth and substrates for macromolecule biosynthesis^2^, including substrates for de novo lipogenesis (DNL), which is necessary for membrane biosynthesis and generation of signaling molecules^3,4^. Evidence suggests that DNL is increased or dysregulated in cancerous tissue as compared to normal tissue^5,6^. Targeting fatty acid synthase (FASN) has shown that breast cancer models are highly-dependent on DNL for growth^7–9^. Although this suggests FASN is an attractive therapeutic target in breast cancer, use of FASN-targeting drugs has been limited by serious side effects^10^. Additional therapeutic targets in the DNL pathway are being investigated and may lead to the development of improved therapeutic strategies^9,11,12^.

Sterol regulatory element binding proteins (SREBPs) are considered master transcriptional regulators of DNL because they control expression of multiple key enzymes in lipid and cholesterol synthesis pathways^13,14^. In general, it is thought that SREBP1, which can be expressed as two splice variants, 1a and 1c, each with different transcriptional activity^13,14^, controls fatty acid synthesis whereas the related family member, SREBP2, controls cholesterol synthesis. As a result, blocking SREBP may be therapeutically viable but this has yet to be examined in breast cancer^15–20^. Here, we explore the therapeutic potential and mechanism of action of the small molecule inactivator of SREBP, called fatostatin (FS)^21^. FS binds to SREBP cleavage-activating protein (SCAP), a critical regulator of SREBP activity^13,21^, to prevent the processing and maturation of SREBPs^22,23^. Studies have shown that FS has anti-tumor effects in both prostate and pancreatic tumor cells through inhibition of SREBP-dependent processes^20,22^. However, FS can also have SREBP-independent activities, such as inhibition of microtubule formation and endoplasmic reticulum protein processing^17,23,24^.

We report that FS inhibits growth and induces apoptosis in estrogen receptor (ER)-positive breast cancer cells and tumors in a SREBP-independent but endoplasmic reticulum stress (EnRS)-dependent manner. Moreover, we find that FS induces global changes in cellular lipid content, despite the lack of effect on SREBP1 maturation or activity. Accumulation of ceramides contributes to the apoptotic effects of FS while accumulation of triacylglycerides (TAGs) containing polyunsaturated fatty acids (PUFAs), appears to be a protective mechanism that limits apoptosis, suggesting inhibition of PUFA-TAG production as a novel therapeutic strategy in breast cancer.

## Results

### Fatostatin inhibits growth of ER+ but not ER− breast cancer cells

**Fig. 1:**
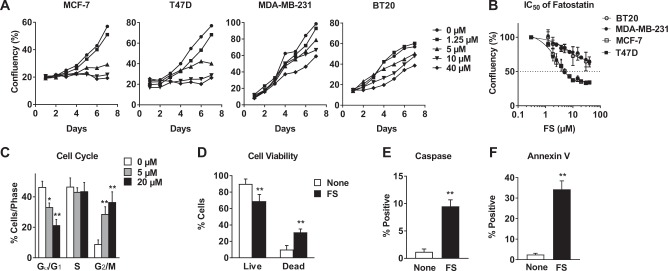
Fatostatin inhibits growth of ER+ cells by arresting cell cycle and activating apoptosis. **a** ER+ (MCF-7 and T47D) and ER− (MDA-MB-231 and BT20) breast cancer cells were treated with FS at doses indicated and cell confluency was measured over 7 days. Media was changed and cells were retreated every 2–3 days. **b** The IC50 (concentration of FS leading to 50% inhibition of the cell growth) was determined based on confluency on day 7. **c** MCF-7 cells were treated with FS for 48 h and cell cycle analysis was carried out using a BrdU assay. **d**–**f** MCF-7 cells were treated with 5 μM FS for 48 h and cell viability was measured (**d**). Apoptosis was measured using caspase 3/7 substrate cleavage (**e**) and Alexa Fluor 488 Annexin V staining (**f**). * *P* < 0.05 vs none; ** *P* < 0.01 vs none

ER positive (MCF-7 and T47D) and negative (MDA-MB-231 and BT20) cell lines were treated with increasing doses of FS and confluency was measured over 7 days (Fig. 1a). FS inhibited cell growth of ER+ cells with an IC_50_ of ~5 μM but was less effective in the ER− cell lines (IC_50_ > 40 μM, Fig. 1b). The reduced growth of ER+ cells was attributed to both cell cycle arrest (Fig. 1c and Supplementary Fig. 1A) and increased apoptosis (Fig. 1d–f, Supplementary Fig. 1B-D). No effect of 5 μM FS on cell viability was observed in MDA-MB-231 cells (Supplementary Fig. 1e).

### Fatostatin promotes accumulation of cellular lipids through endoplasmic reticulum stress

**Fig. 2:**
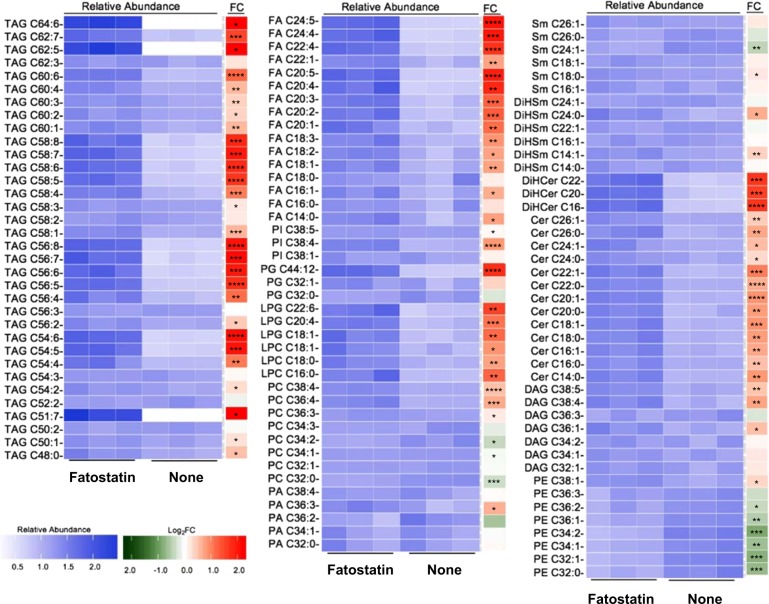
Fatostatin causes accumulation of lipids. The heat map demonstrates the relative abundance and corresponding fold change (log2) of lipids in untreated MCF-7 cells or cells treated with 5 μM FS for 48 h. Significant accumulations in TAG, FA, and ceramide species in FS-treated cells are observed (* *P* < 0.05, ** *P* < 0.01, *** *P* < 0.001, **** *P* < 0.0001)

Since FS is thought to act by inhibiting SREBP activation and DNL, we assessed lipid levels using a global lipidomic approach following FS treatment. We first investigated the lipid composition using untargeted lipidomics to obtain a global view of the changes induced by FS. Surprisingly, rather than a depletion of lipid content, we observed significant accumulation of fatty acids (FAs), TAGs, ceramides, and dihydroceramides upon FS treatment (Supplementary Table 1). We confirmed the overall accumulation of lipids using targeted analysis where we investigated the levels of representative members from different lipid families (Supplementary Table 1). Figure 2 summarizes the results from untargeted and targeted analysis in a heatmap showing relative abundance for each lipid. Notably, the majority of FAs and TAGs accumulated contained PUFAs.

**Fig. 3:**
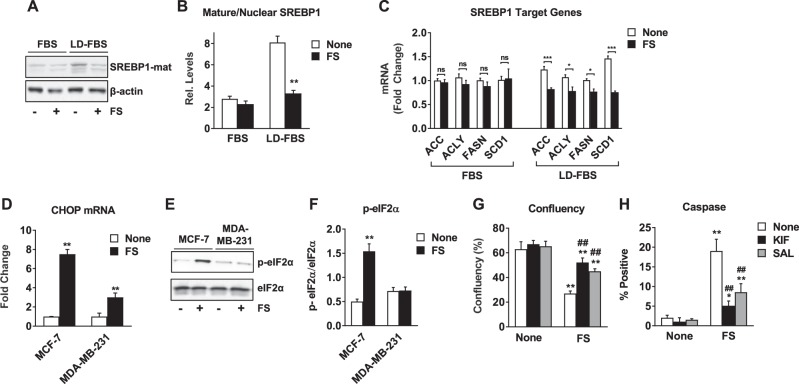
Fatostatin blocks SREBP translocation to the nucleus in lipid-depleted conditions but induces EnRS in lipid-containing conditions. **a**, **b** MCF-7 cells were grown in media supplemented with 10% FBS or 10% lipid-depleted FBS (LD-FBS) for 24 h prior to being treated with 5 μM FS for 24 h. Nuclear extracts were prepared and the mature form of SREBP1 was detected by Western Blot. β-actin served as a loading control. **b** Quantitation of mature/nuclear SREBP1 relative to β-actin is indicated based on analysis of Western blots. **c** RT-QPCR was performed for SREBP1 target genes. **d–f** Indicated cell lines were treated with 5 μM FS in 10% FBS for 48 h. RT-QCR was performed for C/EBP homologous protein (CHOP) mRNA (**d**). Phospho and total eIF2α were detected by Western Blot and the ratio was quantified (**e**, **f**). **g**, **h** MCF-7 cells were treated with 5 μM FS for 48 h in the presence or absence of the EnRS inhibitors salubrinal (SAL, 50 μM) or kifunensine (KIF, 1 μM). Confluency and caspase activity were measured as in Fig. 1. * *P* < 0.05 vs none; ** *P* < 0.01 vs none; ## *P* < 0.01 vs FS alone; ns not significant

Given the unexpected accumulation rather than depletion of lipids in response to FS, we investigated the effect of FS on SREBP activity. When studies were conducted in 10% FBS, as was the case for results shown in Figs. 1 and 2, FS had little effect on SREBP1 nuclear localization or target gene expression (Fig. 3a–c). In contrast, FS inhibited SREBP1 when studies were conducted in 10% lipid-depleted FBS (LD-FBS) (Fig. 3a–c). No changes were observed in cholesterol or cholesterol ester levels, or the SREBP2 target gene, 3-hydroxy-3-methylglutaryl-CoA reductase (data not shown), in response to FS in media containing 10% FBS, suggesting that FS is not inhibiting SREBP2 activity either. These findings suggest that FS can inhibit SREBP function in lipid-depleted conditions where SREBP is active, but can act through a SREBP-independent mechanism when lipids are available in the environment.

Previous studies have shown that FS affects not only SCAP/SREBP activity but also protein translocation from the endoplasmic reticulum to the Golgi^23^, so we asked whether FS might cause EnRS in breast cancer cells. We found that CHOP mRNA and phosphorylated eIF2α, classical markers of EnRS, were up-regulated by FS in ER+ but not in the ER− cells (Fig. 3d–f, Supplementary Fig. 2A). To determine if EnRS contributed to apoptosis, we utilized kifunensine and salubrinal to inhibit EnRS and found that both substantially reversed the effect of FS on cell growth and apoptosis (Fig. 3g, h and Supplementary Fig. 2b, c). Interestingly, thapsigargin, a known inducer of EnRS also had little effect on growth of ER− cells (Supplementary Fig. 2D) but mimicked the growth inhibitory and proapoptotic effect of FS in ER+ cells (Supplementary Fig. 2e, f). These findings suggest that the ability of FS to induce EnRS may underlie the cell-selective responses to FS.

**Fig. 4:**
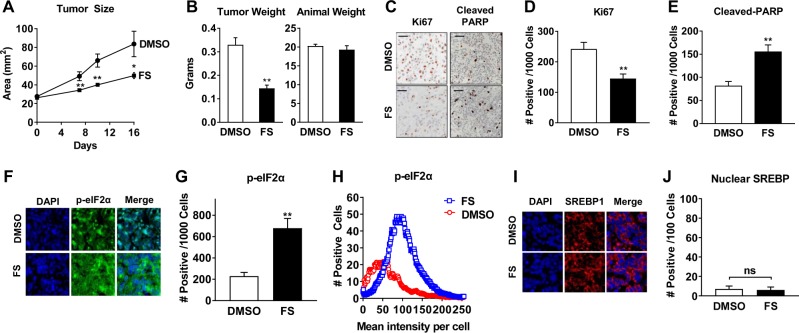
Fatostatin inhibits growth of and induces EnRS in MCF-7 xenograft tumors. MCF-7 cell xenograft tumors were initiated in athymic mice supplemented with estradiol capsules. Once tumors reached ~25 mm^2^, FS or DMSO control were administered daily (*n* = 12–14 tumors/group). **a** Tumor size was measured and plotted over time. **b** Tumors were excised after 16 days of treatment and weighed. Animal body weight on day 16 is indicated. **c–e** Ki67 and cleaved PARP were examined in FFPE tumor sections by IHC and quantified. Bars represent 100 µm. **f** p-eIF2α was examined by IF with DAPI as a nuclear stain in DMSO and FS-treated tumors. **g** Quantitation of immunofluorescence was performed using the cell seed/spot segmentation analysis in ImageJ FIJI. The number of cells with color intensity +5% over background were counted as positive staining for p-eIF2α. **h** The mean color intensity of each cell staining positive for p-eIF2α was determined and plotted as number of cells vs. intensity. **i** SREBP1 was examined by IF with DAPI as a nuclear stain in DMSO and FS-treated tumors. **j** The number of nuclei with SREBP1 staining was determined and plotted per 100 cells. * *P* < 0.05 vs DMSO control; ** *P* < 0.01 vs DMSO control

The growth inhibitory effect of FS was confirmed in vivo using MCF-7 cell xenograft tumors initiated in the mammary glands of mice. Treatment of mice with FS for 16 days decreased growth of xenografts as compared to DMSO control (Fig. 4a). End point measurements showed a 50% reduction of tumor weight in FS-treated mice (Fig. 4b). However, no change in body weight was observed (Fig. 4b). Inhibition of proliferation and activation of apoptosis in xenograft tumors by FS was shown by IHC staining of Ki67 and cleaved PARP, respectively (Fig. 4c–e). We also examined the mechanism of FS action in tumors and observed a significant increase in EnRS based on p-eIF2α levels (Fig. 4f–h), but no change in nuclear SREBP1 levels (Fig. 4i, j). These findings are in agreement with cell culture experiments and suggest that FS may act through induction of EnRS rather than SREBP inhibition to reduce tumor growth and induce apoptosis.

### Fatostatin-induced ceramide synthesis contributes to cell death

**Fig. 5:**
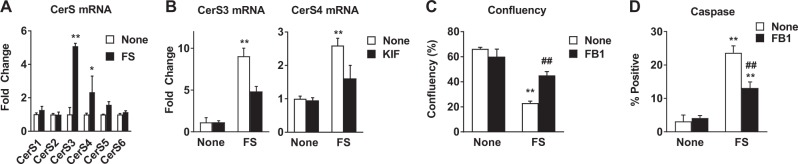
Fatostatin induces apoptosis through ceramide synthase regulation and ceramide production. **a** Expression of ceramide synthase genes in MCF-7 cells was determined by RT-QPCR following treatment with FS for 48 h. **b** *CerS3* and *CerS4* mRNA were measured in MCF-7 cells treated with FS in the presence or absence of the EnRS inhibitor KIF. **c**, **d** Confluency and caspase activity were measured in FS treatment MCF-7 cells in the presence or absence of the ceramide synthase inhibitor FB1 (5 μM). *P < 0.05 vs none; **P < 0.01 vs none; ##*P* < 0.01 vs FS alone

Of particular interest from lipidomic profiling was the accumulation of dihydroceramides and ceramides (Fig. 2, Supplementary Table 1) due to the established connection between ceramides and apoptosis^25^. We compared ceramide and dihydroceramide levels in MCF-7 cells in culture, MCF-7 xenograft tumors, and MDA-MB-231 cells and found that FS induced a significant increase in dihydroceramides and ceramides in ER+ cells and xenografts but not in ER− cells (Supplementary Fig. 3A). Next, we investigated whether FS affected the expression of ceramide synthases (*CerSs*) and found a significant increase in *CerS3* and *CerS4* mRNA in MCF-7 cells treated with FS (Fig. 5a) but not in ER− MDA-MB-231 cells (Supplementary Fig. 3B). Similar results were observed in additional cell lines (Supplementary Figs. 3C and 3D). Importantly, the EnRS inhibitor kifunensine blocked the up-regulation of *CerS3* and *CerS4* expression (Fig. 5b) after FS treatment. Next, we used a CerS inhibitor, Fumonisin B1 (FB1), to evaluate the role of ceramide synthesis in FS-treated ER+ cells. We found that FB1 does not have any effect on cell growth or survival in the absence of FS but significantly reversed the effect of FS on cell confluency and apoptosis (Fig. 5c, d and Supplementary Figs. 3E, 3F). These findings indicate that the ability of FS to induce apoptosis is mediated in part through the up-regulation of CerSs and production of dihydroceramides and ceramides, and that this appears to be a consequence of FS-induced EnRS stress.

### Fatostatin-induced PUFA-TAG accumulation is a protective mechanism that contributes to cell survival

**Fig. 6:**
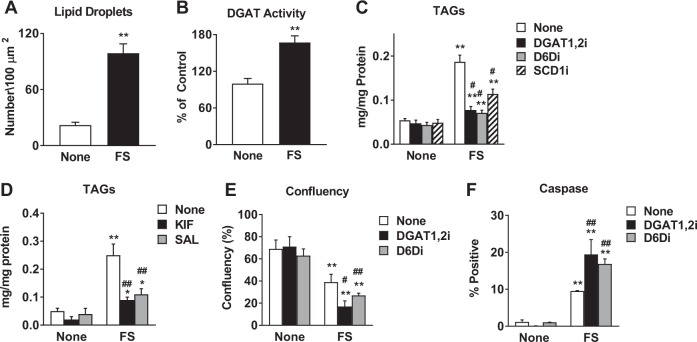
Accumulation of PUFA-TAGs plays a protective role. **a** Nile Red staining was performed on MCF-7 cells treated with 5 μM FS for 48 h. The number of lipid droplets per 100 µm^2^ were quantified by ImageJ FIJI. **b** DGAT activity was measured in untreated and FS-treated MCF-7 cells, as described in Materials and Methods section. **c** TAGs were measured using Triglyceride Colorimetric Assay Kit in MCF-7 cells treated with FS in the presence or absence of DGAT inhibitors (DGAT1,2i, 10 μM each), Δ6 desaturase inhibitor (DGDi, 50 μM), or SCD inhibitor (SCDi, 50 μM). **d** TAGs were measured in untreated or FS-treated MCF-7 cells in the presence or absence of EnRS inhibitors (KIF, 1 μM or SAL, 50 μM). **e**, **f** Confluency and caspase activity were measured following treatment with FS in the presence or absence of DGAT1,2i or D6Di. * *P* < 0.05 vs none; ** *P* < 0.01 vs none. # *P* < 0.05 vs FS alone; ## *P* < 0.01 vs FS alone

In parallel to the accumulation of ceramides and dihydroceramides, the untargeted lipidomics analysis highlighted the accumulation of PUFA-TAGs (Fig. 2). We first confirmed that FS induces an accumulation of lipid droplets, which contain neutral lipids such as TAGs, by Nile Red staining (Fig. 6a, Supplementary Fig. 4A). We also observed an increase in total TAG content in ER+ but not ER− breast cancer cells treated with FS (Supplementary Fig. 4B). Next, to understand how TAGs accumulate during FS treatment, we investigated key enzymes in TAG synthesis, diacylglycerol acyltransferase 1 and 2 (DGAT1,2). We found that DGAT1,2 activity increased by 70% after FS treatment in MCF-7 cells (Fig. 6b) and that inhibition of DGAT1,2 significantly decreased accumulation of TAGs, indicating that DGAT activity is involved in the accumulation of TAGs (Fig. 6c, Supplementary Fig. 4C). We also observed that kifunensine and salubrinal also prevented TAG accumulation in response to FS (Fig. 6d), indicating that TAG accumulation is also a consequence of EnRS.

The specific accumulation of PUFA-TAGs suggests that desaturases may be involved in TAG accumulation during FS treatment (Fig. 2). In fact, inhibition of desaturases responsible for PUFA production, Δ-6-desaturase (D6D) and stearoyl-CoA desaturase-1 (SCD1), also inhibited overall TAG accumulation (Fig. 6c, Supplementary Fig. 4C). Functionally, we found that inhibition of DGAT1,2 or D6D had little effect on their own but significantly enhanced the ability of FS to inhibit cell growth and promote apoptosis (Fig. 6e, f and Supplementary Figs. 4D, 4E). These findings suggest that FA desaturation is a key step in promoting TAG synthesis and accumulation. Based on these results, we propose that the incorporation of PUFAs into TAGs can protect cancer cells from FS-induced apoptosis. This mechanism also appears to be specific to FS action in ER+ breast cancer cells and a consequence of the ability of FS to induce EnRS.

## Discussion

**Fig. 7:**
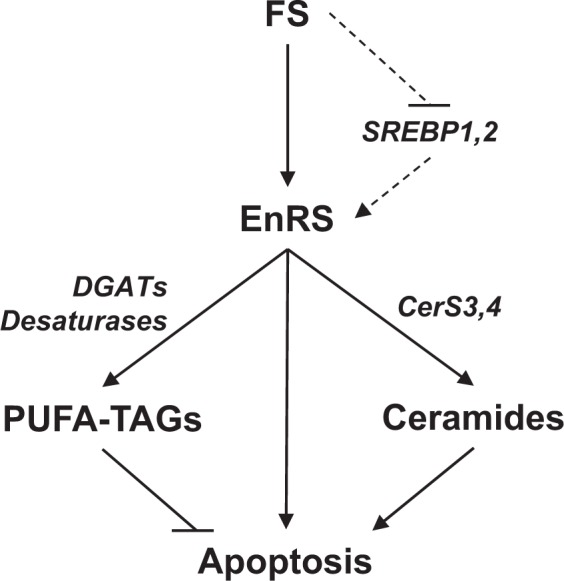
Model summarizing novel anti-cancer mechanisms of action of FS in ER+ breast cancer cells. Solid lines represent data presented in Figs. 1–6 while dotted lines represent a hypothetical mechanism by which FS may induce EnRS

Together, our findings suggest that FS acts to inhibit growth and induce apoptosis in ER+ breast cancer cells and tumors. The mechanism of FS action in lipid-sufficient conditions involves activation of EnRS and the accumulation of lipid species (Fig. 7). The source of lipids for this lipid accumulation is unclear but de novo lipogenesis genes, such as FASN and ACC, are not regulated (either up or down) by FS in lipid-containing FBS suggesting other lipogenesis regulators, such as ChREBP or LXRα, are not involved^26^. SCAP/SREBP inhibition does not appear to be involved in FS action in these conditions as SREBP target genes were not regulated and lipid levels were not reduced. However, SCAP/SREBP inhibition has been linked to induction of EnRS^18^, making this pathway a potential contributor to the findings we have observed. Both pro-apoptotic and anti-apoptotic lipid species accumulated in response to FS. On one hand, ceramide synthesis was elevated, which contributed to the apoptotic effects of FS, while on the other, FS activated DGATs and promoted accumulation of PUFA-TAGs, which appeared to play a protective role and limit apoptosis. Overall, global changes in the lipidome in response to FS tended to favor cell death but this could be further enhanced by blocking PUFA-TAG production.

Fatostatin is primarily thought to block the activation process of SREBPs and thereby inhibit lipogenesis. We confirmed that in lipid-deprived conditions FS blocks accumulation of nuclear SREBP1. However, there was no effect of FS on nuclear SREBP1 in cells grown in lipid sufficient conditions. It is also important to note that in xenograft tumors, FS did not appear to affect nuclear SREBP1, suggesting that there are sufficient lipids present in the mammary gland tumor microenvironment to keep SREBP signaling suppressed. In spite of the absence of SREBP inhibition, FS was still able to block ER+ breast cancer cell and tumor growth. While it is possible that systemic effects of FS on liver and adipose lipid metabolism may contribute to reduced tumor growth, we observed no effect on body weight over the 16 days of treatment. It should be noted that previous studies on the metabolic effects of FS were observed in the ob/ob mouse model of obesity and did not become evident until after 20 days of treatment^21^. Moreover, the increase in EnRS and ceramide levels was observed both in vitro and in vivo, suggesting the inhibitory effects of FS on tumor growth may be direct. In agreement with our work, others have shown that FS-inhibited cell growth is not always rescued by the addition of exogenous lipids and that FS can inhibit growth of cells lacking SCAP^23^. Alternative mechanisms of FS action have been suggested and include the inhibition of tubulin polymerization^17^, general EnR-to-Golgi transport^23^, and androgen receptor activity^22^. Our studies suggest that activation of EnRS is one of the major mechanisms by which FS influences ER+ breast cancer cells.

Throughout our studies, we showed several major differences in cell type-selective responses to FS. While our findings that FS-induced apoptosis, EnRS, ceramide synthesis, and PUFA-TAG accumulation in ER+ but not in the ER− cells suggest that the effects of FS may be selective for ER+ cells, it is also possible that FS action is dependent on whether cells respond to FS with EnRS or not, which may also be related to ER status. Studies have shown that an indole-3-carbinol derivative up-regulates key signaling molecules involved in EnRS to a much greater extent in MCF-7 than MDA-MB-231 cells^27^. It was also shown that the expression of lipin 1, an important regulator of FAs, is elevated in triple negative breast cancer cells and prevents EnRS through regulation of phospholipid synthesis^28^.

Previous studies indicate that activation of EnRS can induce ceramide production^25,29^ and that ceramides themselves can trigger EnRS^30,31^. Our studies suggest that inhibition of EnRS partially blocked *CerS* transcription, suggesting that EnRS lies upstream of ceramide production. Additionally, we and others have shown that accumulation of PUFA-TAGs also appear to be downstream of EnRS^25,32^. Genetic depletion of PERK, an endoplasmic reticulum stress-regulated kinase, was shown to attenuate cholesterol, PUFA, and TAG biosynthesis^33^. While the apoptotic effects of ceramide synthesis are well-established^25,29^, the accumulation of PUFA-TAGs as an anti-apoptotic or protective mechanism is only now being investigated^31,34^. It was previously shown that accumulation of saturated FAs, in contrast to unsaturated FA, resulted in activation of apoptosis and cell death^35,36^. Interestingly, Williams et. al. have shown that desaturation can be uncoupled from lipogenesis when SCAP/SREBP activity is inhibited in cancer cells^37^ which may be consistent with fatostatin action in lipid-depleted conditions. Thus, scavenging of FAs through desaturation and TAG production may represent a mechanism by which cancer cells protect themselves against FA-induced lipotoxicity and apoptosis^31,38^.

In conclusion, the data presented herein reveal a mechanism by which FS affects breast cancer cell and tumor growth. This includes activation of EnRS, up-regulation of CerSs, and the accumulation of ceramides that triggered apoptotic cell death. We showed that EnRS also activates PUFA-TAG accumulation, which partially protects cancer cells against saturated FA lipotoxicity. Moreover, we showed that FS affects ER+ cancer cells and xenografts but not ER− cancer cells as a consequence of EnRS. These studies provide proof-of-principle evidence that treatment of ER+ cancer with FS could provide a viable therapeutic approach independently of SREBP inhibition. Finally, our studies suggest that combination of FS with inhibitors of PUFA-TAG biosynthesis could provide a novel and efficient approach for breast cancer therapy.

## Materials and methods

### Reagents

FS was purchased from Tocris. Kifunensine, Salubrinal, FB1, and DGAT inhibitors were purchased from Sigma. Thapsigargin and D6D and SCD1 inhibitors were purchased from Cayman. Antibodies against Ki67 and p-eIF2α (#9027S; #3398S) were purchased from Cell Signaling Technology. Antibodies against SREBP1, PARP, and cleaved-PARP were purchased from Abcam (#ab28481; #ab32138; #ab32064; #ab150064; #ab150077).

### Cell lines and cell culture

Human MCF-7 cells were obtained from Dr. Donald McDonnell (Duke University). T47D and BT20 cell were provided by Dr. Debra Tonetti (UIC) and Dr. James Radosevich (UIC). MDA-MB-231 cell were received from Dr. Clodia Osipo (Loyola University). All cell lines were maintained in Roswell Park Memorial Institute (RPMI) media supplemented with 10% fetal bovine serum (FBS), 1% non-essential amino acids, 2 mM L-glutamine, and 1% penicillin-streptomycin at 37 ˚C in 5% CO_2_. Cell line authentication is routinely performed using short tandem repeat methodology. All cell lines are routinely evaluated for mycoplasma contamination. Delipidation of serum was performed by adding fumed silica to FBS followed by mixing overnight, centrifugation, and filtration.

### Proliferation and apoptosis assays

Cell confluency, viability, apoptosis and cell cycle analyses were performed using a Celigo Image Cytometer (Nexcelom Bioscience). Cell cycle analysis was performed following BrdU incorporation and antibody labeling, as well as DAPI staining^39^. Caspase activity was measured by ViaStain™ Live Caspase 3/7 Detection kit (Nexcelom). FITC-labeled Annexin V binding to phosphatidylserine (PS) was used to evaluate apoptotic cells, and PI staining was used to exclude necrotic cells from apoptotic count. Results were expressed as a percent of Caspase or Annexin V positive cells relative to number of total cells. Cell viability was measured with ViaStain™ Calcein AM/PI/Hoechst Cell Viability Kit (Nexcelom).

### Western blotting

Whole cell extracts were prepared using RIPA buffer; proteins were denatured and separated by SDS-PAGE and then transferred to nitrocellulose membranes. Membranes were blocked in non-fat dry milk or BSA and incubated overnight with primary antibodies. Membranes were then washed and incubated with secondary antibodies. The signal was visualized using Chemi-doc XRS (Bio-Rad Laboratories) and the Pierce Supersignal West Pico Chemiluminescent Substrate (Thermo Scientific).

### RNA and RT-QPCR

Total RNA was isolated using Trizol according to the manufacturer's instructions (Ambion). RNA was isolated and RT-QPCR performance as previously published^40^. Primer sequences are available upon request. Fold change was calculated using the ΔΔCt method. 36B4 or GAPDH was used as the internal control.

### Xenograft tumors

Experiments were carried out at the UIC Biological Resources Laboratory and all procedures and studies were approved by the Animal Care and Use Committee according to institutional and national guidelines. MCF-7 cell xenografts were established by injecting 5 million cells suspended in matrigel into the mammary glands of five week old athymic female nude mice (nu/nu, Taconic). Silastic capsules (0.3 cm) containing 17β-estradiol (Sigma) were implanted at the same time as cell injection. When the average tumor size reaches 25 mm^2^, animals received FS (30 mg/kg/day) or DMSO by intraperitoneal (IP) injection. After 16 days of treatment, mice were humanely euthanized by CO_2_ inhalation followed by cervical dislocation and tumors were extracted and weighted. Half of each tumor was fixed for immunohistochemistry and immunofluorescence studies. The other half was flash frozen in liquid nitrogen until subsequent biochemical analysis of ceramides.

### Immunohistochemistry and immunofluorescence

For immunohistochemistry staining, formalin-fixed, paraffin embedded tissue sections were sectioned at 5 μm and then deparaffinized. Microwave-enhanced antigen retrieval was performed by boiling the slides in 10 mM citrate buffer. After washing, slides were incubated in hydrogen peroxide followed by normal goat serum, and then primary antibodies overnight. After washing, slides were incubated with secondary antibodies and then the sections were stained with 3,3′-Diaminobenzidine and Hematoxylin. A Nikon Eclipse Ti microscope was used for image acquisition. Immunofluorescence staining was conducted following the same procedure except microwave-enhanced antigen retrieval was performed by boiling the slides in DAKO buffer. After washing, the sections were stained with 4′,6-diamidino-2-phenylindole (DAPI). A Zeiss LSM710 microscope was used for immunofluorescence image acquisition. The percentage of cells staining positive for Ki67, cleaved-PARP, SREBP1, and p-eIF2α was determined using ImageJ software.

### Lipid extraction from cells and xenograft tumors

The cellular lipid extraction procedure was adapted from previous protocols^41^. For each experimental condition, three biological replicates of approximately 10^6^ cells were cultured and analyzed. To assist in homogenizing the tumor tissue, metal beads were added to tissue sample, which were freeze-dried. The lyophilized samples were ground using a Mixer Mill MM 400 (Retsch). They were normalized based on dry weight. The samples were resuspended in 1 mL of PBS and sonicated. The tissue suspension was homogenized and vacuum dried as described above for cellular lipid extraction. The dried samples were resuspended in chloroform based on dry tissue weights.

### LC-MS acquisition

LC-MS data acquisition was performed using an Agilent 1260 HPLC with an Agilent 6530 Accurate-Mass QToF mass analyzer. The LC method was adopted from Saghatelian et al. 2004^42^ as described previously^41^ except a 60 min gradient was used for separations.

### Untargeted and targeted lipidomics data analysis

For untargeted lipidomics of MCF-7 cells, three biological replicates of each experimental condition (control and FS-treated), were analyzed in both positive and negative electrospray ionization modes. The raw data were uploaded into MassHunter Profinder (Agilent) where peak alignment was carried out according to unique peak and isotope features. Aligned data from Profinder was then imported into Mass Profiler Professional (Agilent) for statistical analysis. The species were filtered by their frequency with which they appeared in samples of the same condition. Significance was then determined using a student’s *t*-test with all species with *P* ≥ .05 (with Benjamini Hochberg FDR correction) eliminated and the remaining species were filtered based on fold change. Fold change was determined as [abundance_fatostatin-treated_]/[abundance_control_] for each species. Species that were reproducible in two independent profiling experiments with an average fold change greater than or equal to two were identified. The resulting species were individually examined and their abundances re-integrated if necessary. Resulting *m/z*’s were then analyzed by tandem mass spectroscopy.

Targeted analysis of representative members of different lipid families was performed using MassHunter Qualitative Analysis (Agilent). The chromatogram corresponding to the *m/z* for each lipid species of interest was extracted and the peak area manually integrated. Fold change was determined as [abundance_fatostatin-treated_]/[abundance_control_] for each species. Targeted analysis was carried out in one of the two independent profiling experiments where *n* = 3 for each condition.

### Neutral lipid analysis

Nile red staining was performed on cells grown on coverslips, fixed with paraformaldehyde, and incubated with Nile Red (Sigma, 1mg/ml). Stained samples were mounted on slides and Nile Red staining was evaluated under a fluorescent microscope (excitation 520 nm; emission 600 nm). Quantitation of lipid droplets was performed using ImageJ FIJI. For triglyceride measurements, the Triglyceride Colorimetric Assay Kit (Cayman Chemical) was used with minor modifications. Briefly, control and treated cells were collected and lysed, and then treated with the mixture of lipoprotein lipase, glycerol kinase, glycerol phosphate oxidase, and peroxidase with 4-aminoantipyridine and N-ethyl-N-(3-sulfopropyl)-m-anisidine. The resulting color was measured at 540 nm. To exclude free glycerol, a lipoprotein lipase inhibitor (GSK 264220A, Tocris) was used.

### DGAT activity

The DGAT activity assay was adopted from previous protocols^41,43^.

### Statistics

All data are presented as the mean +/− SEM from 3 to 6 independent samples. Statistical analyses were performed using GraphPad Prism software. One-way or two-way ANOVA (followed by Tukey or Sidak posttest) were used where appropriate. All *P* values were two-sided unless otherwise specified.

## Electronic supplementary material

Supplemental Figure Legends

Supplemental Figures

Supplemental Table
