# Supplementary material for: Fatostatin induces pro- and anti-apoptotic lipid accumulation in breast cancer
Source: Oncogenesis. 2018 Aug 24;7(8):66. doi: 10.1038/s41389-018-0076-0 (PMC6107643; doi:10.1038/s41389-018-0076-0)
Supplement: Supplementary file 1 — Supplemental Figure Legends [file 41389_2018_76_MOESM1_ESM.pdf]

SUPPLEMENTAL FIGURE LEGENDS

Supplemental Fig. 1. Fatostatin inhibits growth by arresting cell cycle and activating apoptosis. A. T47D cells were treated with FS at the indicated concentrations for 24 hrs and cell cycle was measured using a BrdU assay. **B-D**. T47D cells were treated with 5 μM FS for 48 hrs. Calcein-AM was used as a dye for live cells, propidium iodide for dead cells, and Hoechst for total cells (**B**). Apoptosis was measured using caspase substrate cleavage (**C**) and Alexa Fluor 488 Annexin V staining (**D**). **E.** MDA-MB-231 cells were treated with 5 μM FS for 24 hrs and viability was measured using Calcein-AM, propidium iodide, and Hoechst stains. * $P<0.05$ vs none; ** $P<0.01$ vs none.

Supplemental Fig. 2. Fatostatin induces apoptosis through activation of endoplasmic reticulum stress (EnRS). A. Indicated cell lines were treated with 5 μM FS in 10% FBS for 48 hrs. RT-QCR was performed for C/EBP homologous protein (CHOP) mRNA. T47D cells (**B, C**) or MDA-MB-231 cells (**D**) were treated with 5 μM FS for 48 hrs in the presence or absence of the EnRS inhibitors salubrinal (SAL, 50 μM). MDA-MB-231 cells were additionally treated with 1 μM thapsigargin, a known inducer of EnRS (**D**). **(E-F)** MCF-7 (**E**) and T47D (**F**) cells were treated with 1μM thapsigargin in the presence or absence of FS for 48 hrs. Confluency and caspase activity were measured as in Fig. 1. * $P<0.05$ vs none; ** $P<0.01$ vs none; ## $P<0.01$ vs fatostatin alone; ns not significant.

Supplemental Fig. 3. Fatostatin induces cell death through ceramide synthase regulation and ceramide production. A. Targeted lipid analysis showed accumulation of ceramides and dihydroceramides (DHC) in MCF-7 cells and xenograft tumors but not MDA-MB-231 cells following FS treatment. **B.** Expression of ceramide synthase genes in MDA-MB-231 cells was determined by RT-QPCR following treatment with FS for 48 hrs. **C, D.** Regulation of *CerS3* and

CerS4 mRNA was determined in various cell lines treated with FS by RT-QPCR. **E, F.** Confluency and caspase activity were measured in FS treated T47D cells in the presence or absence of the ceramide synthase inhibitor, fumonisin B1 (FB1, 5 μM). * P<0.05 vs none; ** P<0.01 vs none; ## P<0.01 vs fatostatin alone.

Supplemental Fig. 4. Accumulation of PUFA-TAGs plays a protective role in FS-treated cancer cells. A. Nile Red staining was performed on MCF-7 and T47D cells treated with 5 μM FS for 48 hrs. **B.** TAGs were measured in various cell lines using the Triglyceride Colorimetric Assay Kit following FS treatment. **C-E.** TAGs (**C**), confluency (**D**), and caspase activity (**E**) were measured in T47D cells treated with FS in the presence or absence of DGAT inhibitors (DGAT1,2i, 10 μM each), ∆6 desaturase inhibitor (DGDi, 50 μM), or SCD inhibitor (SCDi, 1 μM). * P<0.05 vs none; ** P<0.01 vs none; # P<0.05 vs fatostatin alone; ## P<0.01 vs fatostatin alone.

Supplemental Table 1. Untargeted and targeted MCF-7 lipidomics data. The m/z's, abundances, fold changes, observed adducts and retention times of lipids identified for each sample in untargeted and targeted analysis are provided as a PDF file. The untargeted analysis provides the data from two profiling sets. The p-value of the fold change for each lipid species is provided for the targeted analysis. On the targeted analysis sheet, species that are also present in the untargeted lipidomics analysis are highlighted in gray.
