# Supplementary figures and images for: Fatostatin induces pro- and anti-apoptotic lipid accumulation in breast cancer
Source: Oncogenesis. 2018 Aug 24;7(8):66. doi: 10.1038/s41389-018-0076-0 (PMC6107643; doi:10.1038/s41389-018-0076-0)

# Supplemental Figure 1

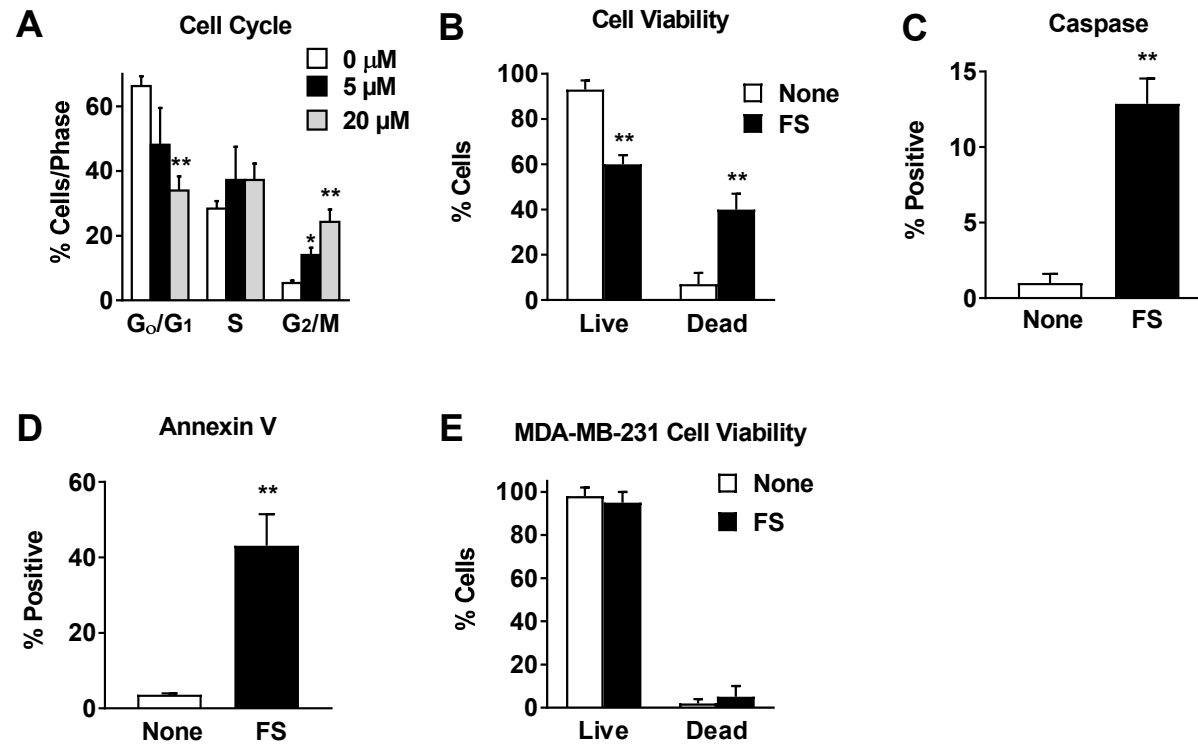

Supplemental Figure 2

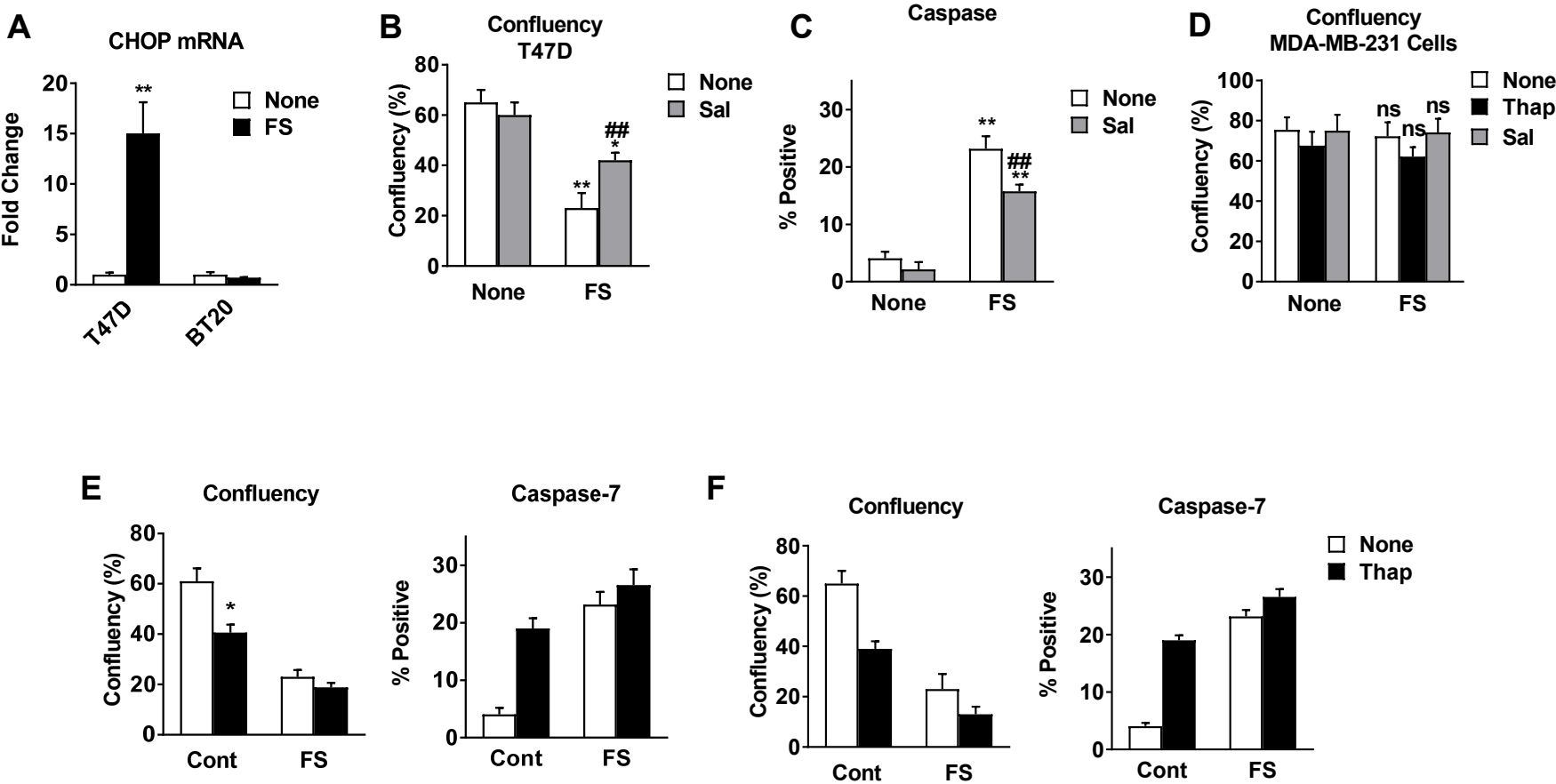

# Supplemental Figure 3

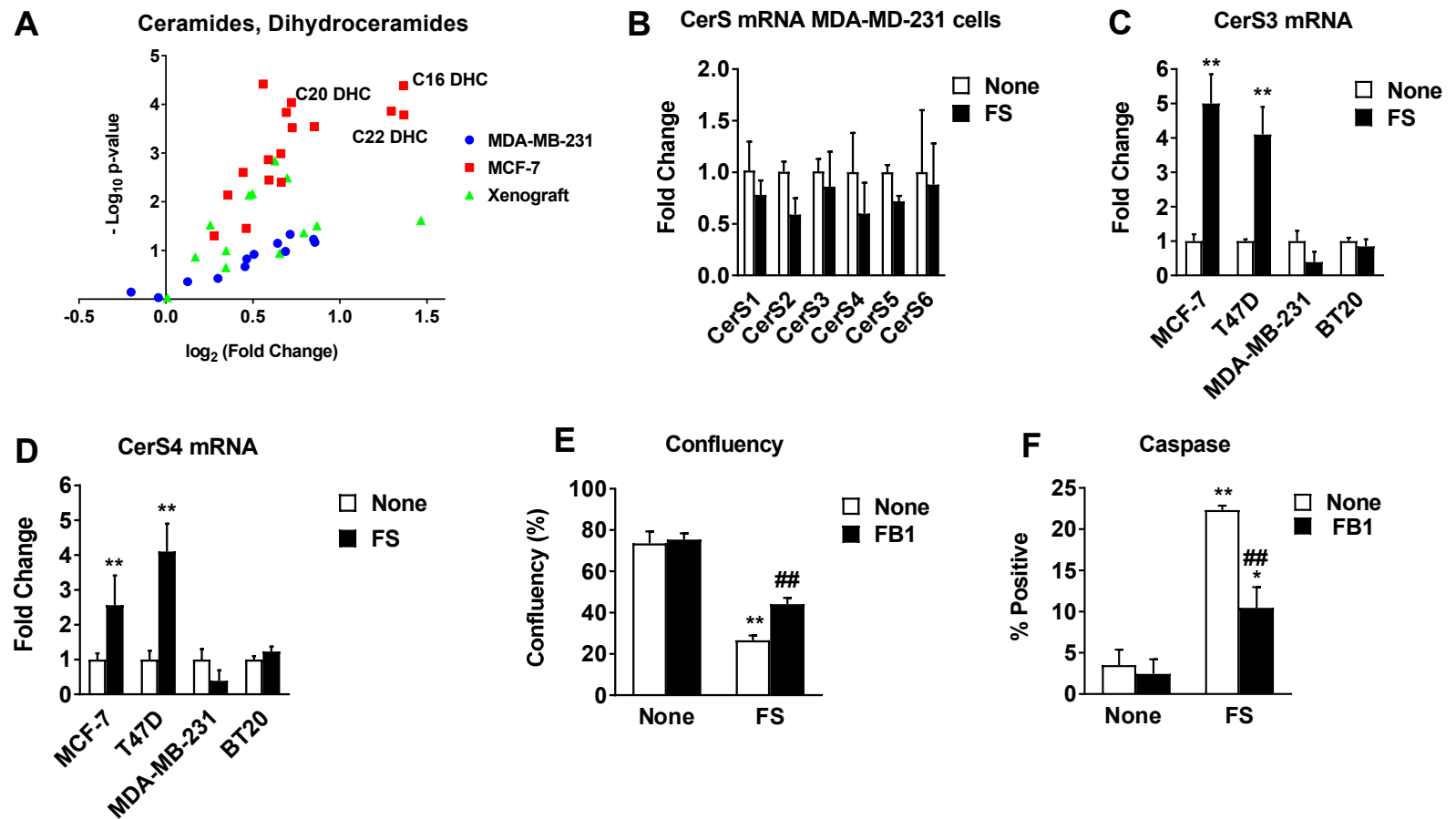

# Supplemental Figure 4

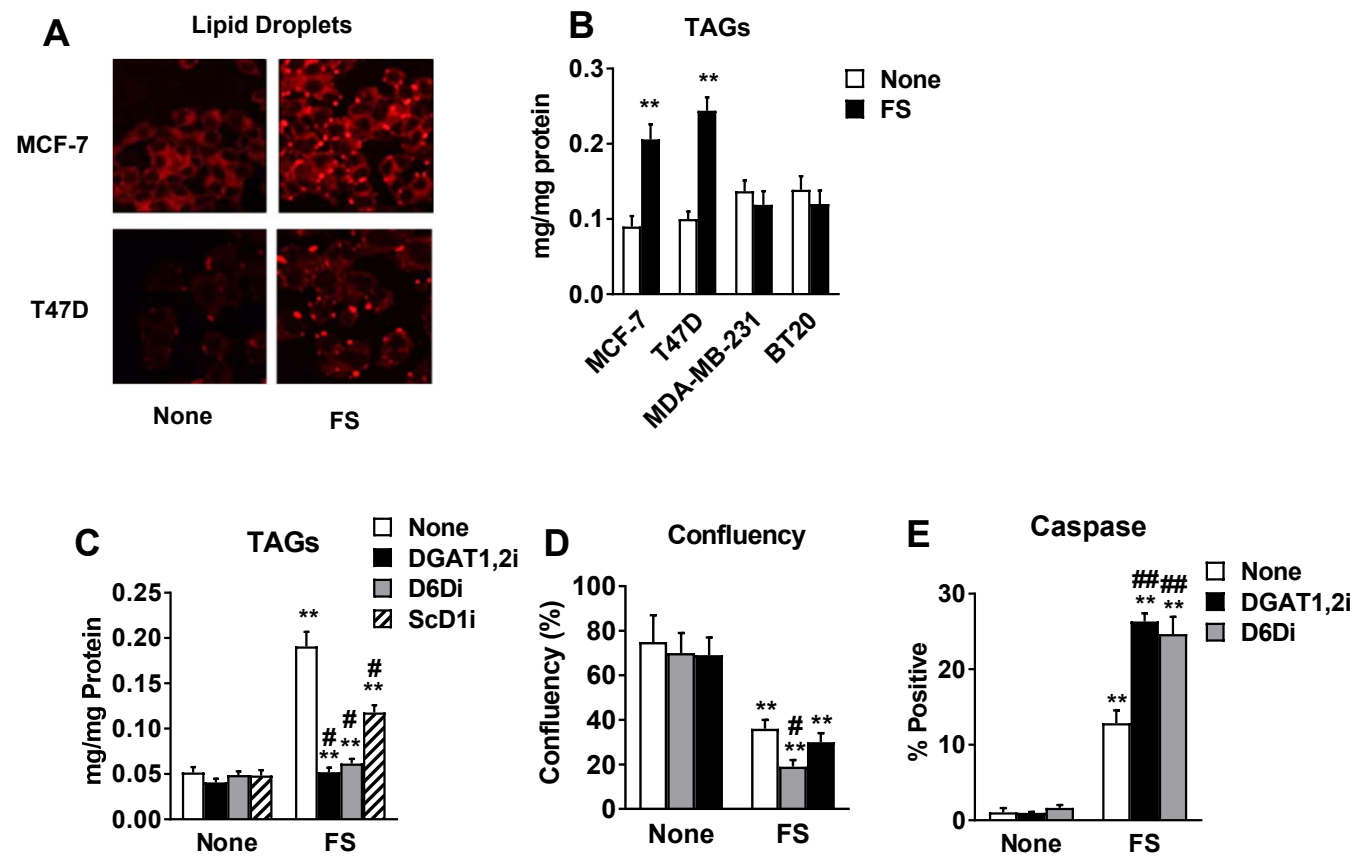

Supplement: Supplementary file 2 — Supplemental Figures [file 41389_2018_76_MOESM2_ESM.pdf]
