# Supplementary material for: Fatostatin induces pro- and anti-apoptotic lipid accumulation in breast cancer
Source: Oncogenesis. 2018 Aug 24;7(8):66. doi: 10.1038/s41389-018-0076-0 (PMC6107643; doi:10.1038/s41389-018-0076-0)
Supplement: Supplementary file 3 — Supplemental Table [file 41389_2018_76_MOESM3_ESM.pdf]

Untargeted lipidomcis results

NEGATIVE	**SET1**									**SET2**									
**lipid**	**m/z**	**R.T. (min)**	**DMSO_1**	**DMSO_2**	**DMSO_3**	**FS_1**	**FS_2**	**FS_3**	**FS/C_1**	**m/z**	**R.T. (min)**	**DMSO_1**	**DMSO_2**	**DMSO_3**	**FS_1**	**FS_2**	**FS_3**	**FS/C_2**	**AVERAGE FOLD CHANGE**
FA(20:2)	307.2646	37.4	587312	742621	577250	1598276	1777438	1465667	2.5	307.2649	37.4	505296	449600	537969	1031601	980452	1124062	2.1	**2.3**
FA(22:4)	331.2656	37.4	258978	354220	327307	1123273	1243706	1034343	3.6	331.2658	37.4	347161	320074	332302	924882	869681	846105	2.6	**3.1**
FA(24:5)	357.2807	38.2	850347	1011806	837034	3219331	3295645	2673792	3.4	357.2805	38.3	771410	774894	728220	2318236	2374211	2514527	3.2	**3.3**
FA(24:4)	359.2957	39.6	239827	257707	202302	819591	772420	674412	3.2	359.2961	39.5	172473	172946	191231	522323	534804	601247	3.1	**3.2**
LPG(22:6)	555.2729	35.5	216930	229596	206979	446467	479321	474963	2.1	555.2734	35.5	193546	250499	256401	514462	536949	626588	2.4	**2.3**
C20 diHCer	594.5841	67.7	125043	122354	141310	343750	309644	326082	2.5	594.585	67.7	196509	152315	148542	418553	395071	407244	2.5	**2.5**
PG(22:6, 22:6)	865.5041	49.3	498997	637876	460557	1663610	1315206	1230028	2.6	865.5047	49.3	477612	448616	460934	1308296	1286121	1168494	2.7	**2.7**

POSITIVE	**SET1**									**SET2**									
**lipid**	**m/z**	**R.T. (min)**	**DMSO_1**	**DMSO_2**	**DMSO_3**	**FS_1**	**FS_2**	**FS_3**	**FS/C_1**	**m/z**	**R.T. (min)**	**DMSO_1**	**DMSO_2**	**DMSO_3**	**FS_1**	**FS_2**	**FS_3**	**FS/C_2**	**AVERAGE FOLD CHANGE**
TAG(16:0,18:1,17:6)	852.7292	64.3	Not detected	Not detected	Not detected	1752471	1606298	1602161	ACCUM	852.7312	63.9	Not detected	Not detected	Not detected	1490103	1266271	1276157	ACCUM	**ACCUM**
TAG(18:0,18:1,20:4)	926.8194	66.8	5004735	6081708	5777987	14462099	12991723	11530120	2.3	926.8218	66.4	4160417	3913553	3907632	11026173	11149864	10352971	2.7	**2.5**
TAG(18:1,18:0,22:6)	950.8164	66.6	2360764	2588163	2614791	5957395	6002000	5855506	2.4	950.8239	66.3	1825744	1898428	1770863	5960373	5929833	5156673	3.1	**2.7**
TAG(18:1,18:1,24:4)	980.8732	67.3	3006021	3828569	3507473	7360603	7291299	7492047	2.1	980.8737	67.1	5270674	5005689	5165589	10343708	10319739	10330029	2.0	**2.1**
TAG(18:1,22:1,22:5)	1006.8929	67.5	1054614	1498608	1452766	3187212	3009213	3039809	2.3	1006.893	67.1	778458	847957	722166	2056442	1773413	1759150	2.4	**2.3**
TAG(18:1,18:0,22:5)	952.8356	66.9	4247657	4334566	4396482	8983927	8614673	8298174	2.0	952.8375	66.6	3059488	3466369	2845353	9178808	8518452	8342914	2.8	**2.4**
TAG(18:0,18:1,22:4)	954.8504	67.2	2020324	2357340	2043779	4976079	4669698	4888957	2.3	954.8536	66.8	2604811	2643786	2563938	6803474	7134154	6249314	2.6	**2.4**
TAG(24:1,18:1, 20:3)	1010.9145	68.1	Not detected	Not detected	Not detected	1819307	1918426	1922366	ACCUM	1010.9123	67.6	Not detected	Not detected	Not detected	988446	1102094	984616	ACCUM	**ACCUM**
TAG(22:4,24:1,18:1)	1036.9281	68.2	Not detected	Not detected	Not detected	1567224	1584278	1634228	ACCUM	1036.9263	67.8	Not detected	Not detected	Not detected	932465	984167	930174	ACCUM	**ACCUM**

Targeted lipidomics results

Gray highlighted species are the results of untargerted analysis

p ≤ 0.05	*
p ≤ 0.01	**
p ≤ 0.001	***
p ≤ 0.0001	****

SET2

Lipid family	Lipid	m/z	Adduct	RT	FS_1	FS_2	FS_3	DMSO_1	DMSO_2	DMSO_3	AVERAGE FS	AVERAGE DMSO	P-VAL	
FAs	C14:0	227.2011	[M - H]-	31.8	18896267	19494459	21834818	15174147	9579152	13957319	20075181	12903539	0.02024	*
	C16:1	253.2168	[M - H]-	33.1	9371845	8090832	9737489	6028844	5095707	7422619	9066722	6182390	0.02649	*
	C16:0	255.2324	[M - H]-	34.9	112854787	103290207	107733736	92037945	67257621	119209682	107959577	92835083	0.37759	
	C18:3	277.2168	[M - H]-	32.8	146327	146069	171403	101491	88616	91890	154600	93999	0.00280	**
	C18:2	279.2324	[M - H]-	34.3	2273752	2398674	2336225	1649580	1159657	1734854	2336217	1514697	0.01087	*
	C18:1	281.2481	[M - H]-	35.8	102794211	91711629	105842671	63909263	54714656	74965391	100116170	64529770	0.00803	**
	C18:0	283.2637	[M - H]-	37.6	81722414	76866346	72353712	65154620	52106258	94823086	76980824	70694655	0.65215	
	C20:5	301.2168	[M - H]-	33.2	838071	741144	808751	282266	260088	262599	795989	268318	0.00006	****
	C20:4	303.2324	[M - H]-	34.6	3497205	3259235	4209832	1222869	1280097	1350679	3655424	1284548	0.00119	**
	C20:3	305.2481	[M - H]-	35.5	942050	999603	1013413	475542	411267	553182	985022	479997	0.00041	***
	C20:2	307.2598	[M - H]-	37.1	1031601	980452	1124062	505296	449600	537969	1045372	497622	0.00037	***
	C20:1	309.2699	[M - H]-	38.1	30601030	28513431	22884739	13896058	16324639	12318516	27333067	14179737	0.00701	**
	C22:1	337.3107	[M - H]-	40.5	3203263	3349202	3515514	2347066	1810183	2366327	3355993	2174525	0.00437	**
	C22:4	331.2656	[M - H]-	36.7	924882	869681	846105	347161	320074	332302	880223	333179	0.00002	****
	C24:5	357.2807	[M - H]-	37.6	2318236	2374211	2514527	771410	774894	728220	2402325	758175	0.00001	****
	C24:4	359.2957	[M - H]-	38.8	522323	534804	601247	172473	172946	191231	552792	178884	0.00012	***
ceramides	C16:1	534.48924	[M - H]-	63.1	16330977	14968294	16798512	10508838	11407753	10061926	16032594	10659506	0.00136	**
	C18:1	562.52056	[M - H]-	64.6	499126	475744	526579	287287	315300	305558	500483	302715	0.00030	***
	C20:1	590.55188	[M - H]-	66.3	1042971	971943	989311	586052	629614	606776	1001408	607481	0.00009	****
	C22:1	618.5832	[M - H]-	67.3	7031208	6240062	6777450	3717929	3530854	3850844	6682907	3699876	0.00029	***
	C24:1	646.61452	[M - H]-	68.9	29117843	26956064	21603081	19484735	18702287	18237430	25892329	18808151	0.03517	*
	C26:1	674.64584	[M - H]-	69.1	29002262	25992646	25444948	20271963	21332366	21277664	26813285	20960665	0.00722	**
	C14	508.47326	[M - H]-	62.4	5653470	5254241	5701988	3342245	3500151	3442465	5536567	3428287	0.00015	**
	C16	536.50458	[M - H]-	64.2	174447406	157811968	179384984	122773225	126142965	127327345	170548119	125414512	0.00248	**
	C18	564.5359	[M - H]-	65.9	9883277	9718689	11022677	6343869	6763751	6264818	10208214	6457479	0.00102	**
	C20	592.56722	[M - H]-	67.0	5580354	5167600	5410548	3043337	3144999	4019022	5386167	3402453	0.00394	**
	C22	620.59854	[M - H]-	67.8	13734714	13061555	13209655	8984210	8981349	9192874	13335308	9052811	0.00004	****
	C24	648.62986	[M - H]-	70.7	54914866	51444918	47280707	45786395	37825663	43199992	51213497	42270683	0.04995	*
	C26	676.66118	[M - H]-	72.0	31196437	34440516	36593025	21253260	21967532	24584429	34076659	22601740	0.00355	**
dihydroceramides	C16	538.52024	[M - H]-	64.6	5032635	4653647	5142836	2000080	1921065	1835138	4943039	1918761	0.00004	****
	C20	594.58288	[M - H]-	67.7	418553	395071	407244	196509	152315	148542	406956	165789	0.00014	***
	C22	622.6142	[M - H]-	69.2	678957	738763	782002	292943	257788	301942	733241	284224	0.00016	***
PIs	C38:5	883.5337	[M - H]-	48.2	3666157	3723614	3764656	3536342	3517360	3658454	3718142	3570719	0.04877	*
	C38:4	885.5493	[M - H]-	48.8	8830690	8691385	9029704	5252842	5389449	5813399	8850593	5485230	0.00007	****
	C38:1	891.5963	[M - H]-	52.5	24155945	19516465	19513632	22286652	18255861	18312887	21062014	19618467	0.51878	
PEs	C32:0	690.5074	[M - H]-	54.1	3535210	4274699	3852902	5602298	5379748	6322774	3887604	5768273	0.00617	***
	C36:3	740.5230	[M - H]-	56.0	3379746	4815816	4381721	4937114	4900405	5459911	4192428	5099143	0.12121	
	C34:2	716.5230	[M + H]+	55.20	10945170	12369346	13349893	20250164	19137388	21098922	12221470	20162158	0.00091	***
	C34:1	718.5387	[M + H]+	55.70	8881045	10230345	10437307	13666008	13735248	15137556	9849566	14179604	0.00319	**
	C36:1	746.5700	[M + H]+	58.90	32911819	38595448	37282097	48291398	46896394	49360961	36263121	48182918	0.00305	**
	C36:2	744.5543	[M + H]+	58.20	47234475	62505493	52456448	65675880	66061340	70298835	54065472	67345351	0.04813	*
	C32:1	690.5074	[M + H]+	56.90	3794461	4286846	4452174	6310465	6363632	6832705	4177827	6502267	0.00084	***
	C38:1	774.6013	[M + H]+	57.93	14797424	15200136	15923817	12794227	11343164	13252887	15307126	12463426	0.01277	*
LPGs	C18:1	509.2879	[M - H]-	36.8	428176	339763	359099	187677	165721	219836	375679	191078	0.00404	**
	C20:4	531.2723	[M - H]-	36.8	165038	168865	159121	78106	86002	99344	164341	87818	0.00036	***
	C22:6	555.2729	[M - H]-	35.5	514462	536949	626588	193546	250499	256401	559333	233482	0.00120	**
PGs	C32:1	719.4863	[M - H]-	48.2	2988124	3062833	2368736	2684327	1678681	2433584	2806564	2265531	0.22134	
	C32:0	721.502	[M - H]-	49	1059766	1054527	1088972	1365425	1107951	1240229	1067755	1237868	0.08620	
	C44:12	865.5041	[M - H]-	49.3	1308296	1286121	1168494	477612	448616	460934	1254304	462387	0.00006	****
	C32:0	647.4652	[M - H]-	45.2	1374599	1049673	887992	1290213	1014007	867742	1104088	1057321	0.81698	
	C34:1	673.4808	[M - H]-	46.4	5478655	4218814	3575847	5223610	4552827	3803345	4424439	4526594	0.88998	

PAs	C36:3	697.4808	[M - H]-	47.2	156989	178674	154892	139370	89274	108685	163518	112443	0.03602	*
	C36:2	699.4965	[M - H]-	47.0	1545367	1471265	1015166	2197106	1787099	1672688	1343933	1885631	0.07793	
	C38:4	723.4965	[M - H]-	46.8	273934	257632	256335	303372	258566	213470	262634	258469	0.88302	
	C48:0	824.7707	[M + NH4]+	62.31	47593830	38669666	40402684	32508084	32712708	28798559	42222060	31339784	0.02254	*
	C50:2	848.7707	[M + NH4]+	64.52	77938585	68535248	77745192	75941990	80243701	74430611	74739675	76872100	0.58124	
	C50:1	850.7864	[M + NH4]+	65.31	111308683	98886776	105228749	88817820	97655681	84722086	105141403	90398529	0.04806	*
	C52:2	876.8020	[M + NH4]+	65.72	116888674	118495523	109826681	118720522	134772143	118250639	115070293	123914435	0.21748	
	C54:6	896.7707	[M + NH4]+	65.70	9935440	9403230	8994879	2685874	3046685	2881117	9444516	2871225	0.00002	****
	C54:5	898.7864	[M + NH4]+	67.33	12103455	12113159	10461487	3189825	3755860	3964366	11559367	3636683	0.00018	***
	C54:4	900.8020	[M + NH4]+	68.12	10374163	10960249	8110058	5271783	5400679	5340168	9814823	5337543	0.00676	**
	C54:3	902.8177	[M + NH4]+	67.22	55175218	53579735	53392551	57176958	50432543	47646415	54049168	51751972	0.47050	
	C54:2	904.8333	[M + NH4]+	66.79	96759803	83616838	88579286	81206982	70252283	74331505	89651976	75263590	0.04486	*
	C56:8	920.7702	[M + NH4]+	65.90	1422330	1350910	1299954	385823	453397	429898	1357731	423039	0.00002	****
	C56:7	922.7858	[M + NH4]+	66.20	9165482	7514133	7844924	1969904	2897322	2420474	8174846	2429234	0.00055	***
	C56:6	924.8020	[M + NH4]+	66.40	11273573	12167454	9948180	3512867	3841962	3533247	11129736	3629359	0.00033	***
	C56:5	926.8171	[M + NH4]+	66.80	10291067	11220174	8218336	3468370	3833243	3581513	9909859	3627709	0.00216	**
	C56:4	928.8328	[M + NH4]+	67.10	7231017	5723618	6413748	3405892	3534729	3055598	6456128	3332073	0.00243	**
	C56:3	930.8484	[M + NH4]+	67.40	34544365	32404478	30861651	33973705	32835332	32301368	32603498	33036801	0.73124	
	C56:2	932.8646	[M + NH4]+	67.60	42720659	36224213	39005403	30660080	29426988	31424399	39316758	30503822	0.01104	*
	C58:8	948.8015	[M + NH4]+	66.10	3256653	2656543	2933379	967212	1183177	959211	2948858	1036533	0.00053	***
TAGs	C58:4	956.8641	[M + NH4]+	67.50	4180508	4032023	4304070	2514727	2345456	2054329	4172200	2304838	0.00028	***
	C58:3	958.8797	[M + NH4]+	67.80	19028963	16729044	17922880	16310355	15765760	15605870	17893629	15893995	0.04561	*
	C58:2	960.8954	[M + NH4]+	68.10	27375805	28087535	31470948	28004067	25733355	24279149	28978096	26005524	0.14866	
	C58:1	962.9110	[M + NH4]+	68.40	17882572	17365995	18549479	12380452	12426895	13081428	17932682	12629591	0.00021	***
	C60:4	984.8954	[M + NH4]+	67.90	3721753	3741482	3354174	2525957	2542965	2632912	3605803	2567278	0.00134	**
	C60:3	986.9110	[M + NH4]+	68.20	18129483	17009169	18726215	15344855	13847066	13944331	17954955	14378751	0.00688	**
	C60:2	988.9267	[M + NH4]+	68.60	27156154	22298774	24757050	20577426	19620134	19726528	24737326	19974696	0.02938	*
	C60:1	990.9423	[M + NH4]+	68.90	11644493	12556570	11727229	8987471	7973432	8555374	11976097	8505425	0.00110	**
	C62:3	1014.9423	[M + NH4]+	68.80	10640435	9206381	10299817	9634582	7852501	8669457	10048878	8718847	0.11915	
	C51:7	852.7312	[M + NH4]+	63.9	1490103	1266271	1276157	Not detected	Not detected	Not detected	1344177	Not detected	<0.05	*
	C56:5	926.8218	[M + NH4]+	66.4	11026173	11149864	10352971	4160417	3913553	3907632	10843003	3993867	0.00001	****
	C58:7	950.8239	[M + NH4]+	66.3	5960373	5929833	5156673	1825744	1898428	1770863	5682293	1831679	0.00013	***
	C60:6	980.8737	[M + NH4]+	67.1	10343708	10319739	10330029	5270674	5005689	5165589	10331159	5147318	0.00000	****
	C58:6	952.8375	[M + NH4]+	66.6	9178808	8518452	8342914	3059488	3466369	2845353	8680058	3123737	0.00006	****
	C58:5	954.8536	[M + NH4]+	66.8	6803474	7134154	6249314	2604811	2643786	2563938	6728981	2604178	0.00009	****
	C62:7	1006.8930	[M + NH4]+	67.1	2056442	1773413	1759150	778458	847957	722166	1863001	782860	0.00047	***
	C62:5	1010.9123	[M + NH4]+	67.7	988446	1102094	984616	Not detected	Not detected	Not detected	1025052	Not detected	<0.05	*
	C64:6	1036.9263	[M + NH4]+	67.8	932465	984167	930174	Not detected	Not detected	Not detected	948935	Not detected	<0.05	*
	C36:3	601.52	[M - OH]+	59.84	9834835	12344870	9731473	11999049	11469219	13517774	10637059	12328681	0.18315	
	C34:2	575.50	[M - OH]+	59.26	840900	919442	763196	859564	637609	902823	841179	799999	0.68306	
	C32:1	549.49	[M - OH]+	47.70	4483392	3993321	4513737	4188437	3209320	4211603	4330150	3869787	0.28230	
DAGs	C34:1	577.52	[M - OH]+	60.10	72634861	64792620	68668581	56172824	68295934	57728911	68698687	60732556	0.14657	
	C36:1	605.55	[M - OH]+	62.00	182729871	150689941	170549567	115949743	136474033	117361787	167989793	123261854	0.01742	*
	C38:5	625.52	[M - OH]+	60.70	51168018	47290656	47910205	34235602	39855810	36054090	48789626	36715167	0.00413	**
	C38:4	627.54	[M - OH]+	61.60	109118440	96833756	109703940	65786430	78551097	61398443	105218712	68578657	0.00526	**
	C16:0	496.34	[M + H]+	43.97	72319264	71552312	91413960	40321109	39579221	37904453	78428512	39268261	0.00390	**
LPCs	C18:1	522.36	[M + H]+	44.67	43762852	35642654	34309162	21570697	27022121	20753873	37904889	23115564	0.01407	*
	C18:0	524.37	[M + H]+	46.69	62531312	53540494	53470501	38801867	33491454	37088215	56514102	36460512	0.00409	**
	C32:1	732.55	[M + H]+	56.03	781267943	804044945	810686176	780196397	854287692	846526732	798666355	827003607	0.32273	
	C32:0	734.57	[M + H]+	56.71	153548162	152664086	159077459	186215277	187669844	192774036	155096569	188886386	0.00028	***
	C34:1	760.59	[M + H]+	57.23	517483460	512505580	502099113	527621205	530422377	534815869	510696051	530953150	0.01538	*
PCs	C34:2	758.57	[M + H]+	56.91	375706586	359730453	384367870	475406903	415276406	501519315	373268303	464067541	0.02673	*
	C34:3	756.55	[M + H]+	55.64	18153311	17959048	18389415	16702795	21771820	22244100	18167258	20239571	0.30857	
	C36:4	782.57	[M + H]+	56.40	40612586	42479257	44564158	27762171	28823462	28968729	42552000	28518120	0.00031	***
	C36:3	784.59	[M + H]+	56.95	60806496	66120434	65827621	59401947	56414713	57445736	64251517	57754132	0.02833	*
	C38:4	810.60	[M + H]+	56.92	15471957	16016010	15236444	11369983	11577002	11683807	15574804	11543598	0.00008	****
	C24:1	815.7001	[M + H]+	60.7	36890287	36825205	40258306	40728293	41596668	36523541	37991266	39616167	0.44803	
	C24:0	817.7157	[M + H]+	61.2	8730541	7218495	7747127	6039845	4756886	5288013	7898721	5361581	0.01183	*
dihydrosphingomyelins	C22:1	787.6688	[M + H]+	59.50	19203533	17317440	16580144	19042923	18700445	17826226	17700372	18523198	0.39338	

dihydrosphingomyelins	C16:1	703.5749	[M + H]+	55.1	179178696	211842112	184257159	201274083	189923458	175296609	191759322	188831383	0.82805
	C14:1	675.5436	[M + H]+	53.65	33391238	31418122	35433074	27411490	27121125	25939186	33414145	26823933	0.00609 **
	C14:0	677.5672	[M + H]+	54.5	31345524	31345222	34524074	34411490	33121125	26839186	32404940	31457267	0.73078
sphingomyelins	C24:1	813.6844	[M + H]+	60.1	152175891	134835745	140597586	190982011	177507619	180276620	142536407	182922083	0.00351 **
	C16:1	701.5592	[M + H]+	54.1	108103680	98981127	85896474	99477494	79517981	93642402	97660427	90879292	0.48182
	C18:0	731.6062	[M + H]+	56.8	5353896	5089794	4798456	4512350	4574387	4362947	5080715	4483228	0.02561 *
	C18:1	729.5905	[M + H]+	55.8	1131921	1285876	1176799	1084478	1163940	1087312	1198199	1111910	0.17627
	C26:1	841.7157	[M + H]+	60.6	94948460	78447778	83014349	77517917	75202032	76614273	85470196	76444741	0.14324
	C26:0	843.7314	[M + H]+	61.6	39349578	33057288	32858262	39852056	42140862	38775411	35088376	40256110	0.09285
